# Association between iron deficiency anemia and subsequent stomach and colorectal cancer diagnosis in Germany

**DOI:** 10.1007/s00432-023-05534-z

**Published:** 2024-01-30

**Authors:** Sarah Krieg, Sven Loosen, Andreas Krieg, Tom Luedde, Christoph Roderburg, Karel Kostev

**Affiliations:** 1 Department of Gastroenterology, Hepatology and Infectious Diseases, University Hospital Duesseldorf, Medical Faculty of Heinrich Heine University Duesseldorf, Moorenstraße 5, 40225 Duesseldorf, Germany; 2 Department of Surgery (A), University Hospital Duesseldorf, Medical Faculty of Heinrich Heine University Duesseldorf, 40225 Duesseldorf, Germany; 3 Epidemiology, IQVIA, 60549 Frankfurt, Germany

**Keywords:** Iron deficiency, Iron deficiency anemia, Cancer, Gastrointestinal cancer, Epidemiology

## Abstract

**Purpose:**

Iron deficiency anemia (IDA) is the most common form of anemia worldwide, resulting in a high burden of disease. Accumulating evidence suggests that IDA is associated with the development of gastrointestinal (GI) cancers.

**Methods:**

Data from the IDA database (IQVIA) of primary care practices in Germany of adult patients first diagnosed with IDA between January 2005 and December 2021 were retrospectively analyzed and compared with a 1:1 propensity score-adjusted cohort without IDA. Study outcomes were first stomach cancer or colorectal cancer (CRC) diagnosis up to 10 years after the index date as a function of IDA.

**Results:**

A total of 122,502 individuals with IDA and 122,502 individuals without IDA were included. The 10-year cumulative incidence of CRC was 1.4% in the IDA patients compared to 0.8% in the cohort without IDA (p < 0.001). Regression analysis revealed a significant association between IDA and subsequent CRC (HR 2.05; 95% CI 1.83–2.30). Stomach cancer was diagnosed in 0.3% of IDA patients compared to 0.2% in the non-IDA cohort during the 10-year follow-up period (p = 0.002). However, this was significant only in the age group > 80 years (HR 2.73; 95% CI 1.60–4.67) and in men (HR 1.90; 95% CI 1.38–2.61).

**Conclusion:**

These findings add to the literature and suggest an association between IDA and GI cancers. The extent to which this association is due to GI bleeding or other pathophysiological processes that may be caused by IDA requires further investigation, particularly experimental studies.

## Introduction

Iron deficiency anemia (IDA) is the most common form of anemia globally, resulting in a high global burden of disease (McLean et al. 2009; WHO 2011). It is caused by decreased availability of iron for erythropoiesis and is defined by the WHO as an iron deficiency (ID)-induced reduction of the hemoglobin (Hb) concentration in the blood below the age- and sex-specific normal values of 12 g/dL in women and 13 g/dL in men (McLean et al. 2009; WHO 2011). Although histologic examination of bone marrow iron stores is the gold standard for the diagnosis of IDA, it is rarely performed in clinical practice. Instead, according to international guidelines, the diagnostic approach is based on the laboratory determination of ferritin and transferrin saturation (TSAT), both of which are decreased (Peyrin-Biroulet et al. 2015; Camaschella 2019). The most common causes of IDA are reduced dietary iron intake, impaired iron absorption, and blood loss (Shokrgozar and Golafshan 2019). In general, the blood loss that causes IDA is slow, chronic, and occult and is not usually combined with active bleeding or hemodynamic abnormalities (Rockey et al. 2020). Iron is a vital trace element and an essential component of many metabolic processes. As such, it has anti-inflammatory and antioxidant properties and is involved in important immune system functions (Muñoz et al. 2007; Zohora et al. 2018; Aksan et al. 2021; Phipps et al. 2021). Iron has also been implicated in cell proliferation and differentiation, gut microbiota and gut health, DNA synthesis and repair, and metabolic degradation of drugs and toxins (Bohnsack et al. Bohnsack and Hirschi 2004; Dostal et al. 2014; Liu and Huang 2015). However, iron imbalances are thought to affect several iron-dependent metabolic functions and are the pathophysiological basis of several diseases (Kumar et al. 2022). Interestingly, evidence suggests that IDA may even be involved in the development, progression and treatment of cancer, particularly in the gastrointestinal (GI) tract (Pfeifhofer-Obermair et al. 2018; Torti et al. 2018; Wang et al. 2018; Jung et al. 2019; Brown et al. 2020).

Remarkably, the prevalence of GI cancer in patients undergoing endoscopy for IDA is estimated to be about 14% (Cook et al. 1986; McIntyre and Long 1993; Rockey and Cello 1993; Kepczyk and Kadakia 1995). However, these studies are biased by strong patient selection, which may overestimate the prevalence of GI cancer in IDA patients. On the other hand, the prevalence of ID in patients with CRC, for example, is high at about 60%, and about 70% of these patients have IDA (Pasricha et al. 2021).

The aim of the present study was to investigate the association between IDA and the incidence of stomach cancer and CRC in a large German cohort using the Disease Analyzer (DA) database (IQVIA).

## Methods

### Database

This retrospective cohort study was based on data from the DA database (IQVIA). This database, which has been used in several previous studies focused on cancer (Schiffmann et al. 2020; Roderburg et al. 2022; Jacob et al. 2023), contains anonymous data on diagnoses, prescriptions, and basic medical and demographic data from the computer systems used in the practices (Rathmann et al. 2018). The database covers approximately 3–5% of all office-based practices in Germany. The sampling method for the DA database uses statistics from German Medical Association to determine the panel design according to specialist group, German federal state, community size category, and age of physician. It has previously been shown that the panel of practices included in the DA database is representative of general and specialized practices in Germany (Rathmann et al. 2018).

### Study population

**Fig. 1:**
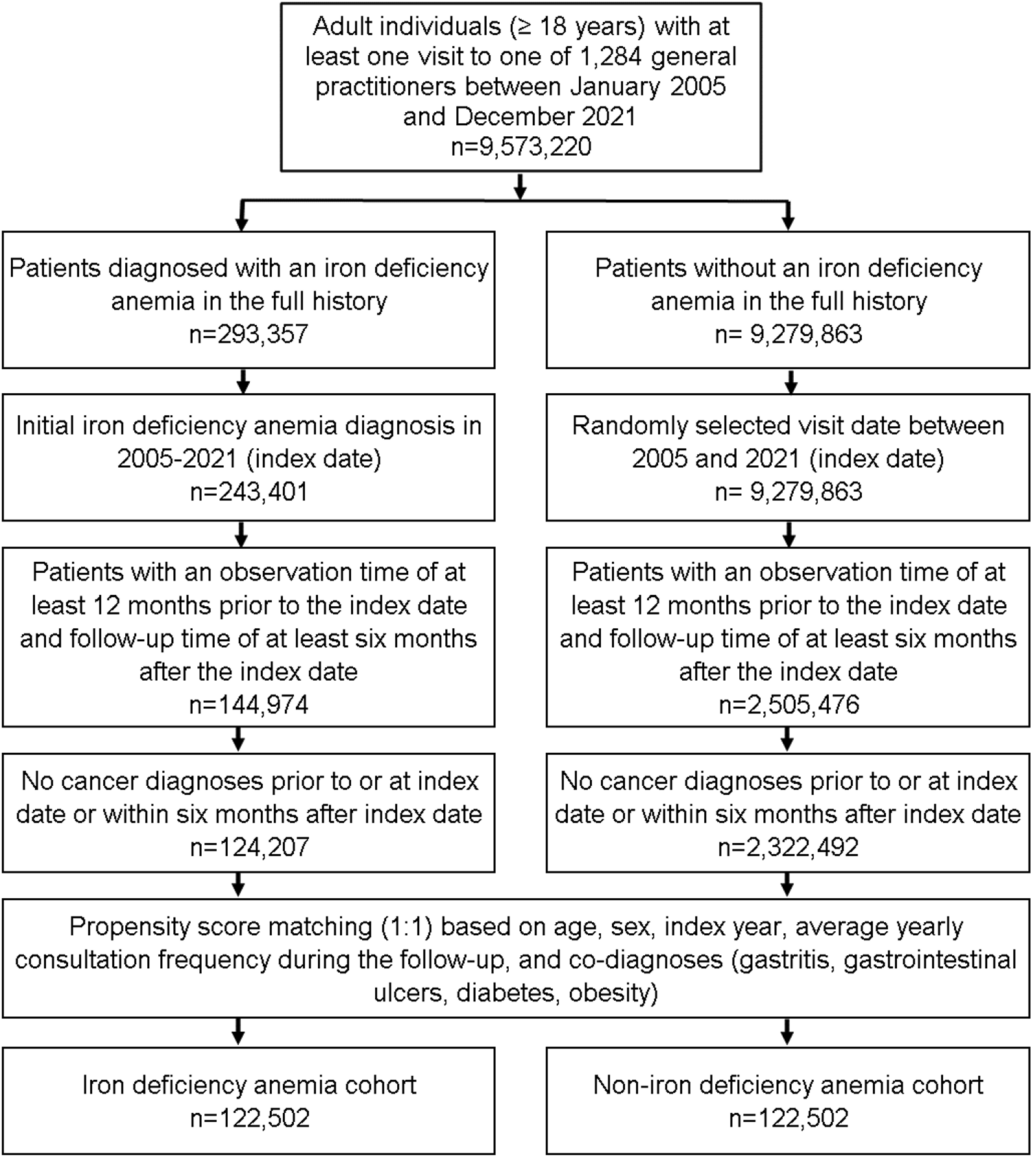
Selection of study patients

This study included patients aged ≥ 18 years with an initial IDA diagnosis (ICD-10: D50) in 1284 office-based general practices (GPs) in Germany between January 2005 and December 2021 (index date; Fig. 1). Further inclusion criterium was an observation time of at least 12 months prior to the index date and a follow-up time of at least 6 months after the index date. Patients with cancer diagnoses prior to or at index date or within 6 months after the index date were excluded. This exclusion criterion was needed to exclude patients with IDA due to bleedings as a symptom of GI cancer. After applying similar inclusion criteria, individuals without IDA diagnoses were matched to IDA patients using nearest neighbor propensity score matching (1:1) based on age, sex, index year, average yearly consultation frequency during the follow-up, and co-diagnoses (GI ulcers (ICD-10: K25-K28), gastritis (ICD-10: K29), diabetes (ICD-10: E10-E14, and obesity (ICD-10: E66)). For the non-IDA cohort, the index date was that of a randomly selected visit between January 2005 and December 2021 (Fig. 1).

### Study outcomes and statistical analyses

The outcomes of the study were the initial diagnoses of CRC (ICD-10: C16, C18) and stomach cancer (ICD-10 C20) in the up to 10 years following the index date as function of IDA. Differences in the sample characteristics and diagnosis prevalence between IDA and non-IDA cohorts were compared using the Wilcoxon signed-rank test for continuous variables, the McNemar test for categorical variables with two categories, and the Stuart–Maxwell test for categorical variables with more than two categories. The 10-year cumulative incidence of cancers in the cohort with and without IDA was further studied with Kaplan–Meier curves, and these curves were compared using the log-rank test. Finally, an univariable Cox regression analysis was conducted to assess the association between IDA and CRC and stomach cancer. Results of the Cox regression model are displayed as hazard ratios (HRs) and 95% confidence intervals (CIs). Additionally, Cox regression analyses were conducted separately for men, and women as well as five age groups. Due to multiple comparisons and large patients samples, a p value of < 0.01 was considered to be statistically significant. Analyses were carried out using SAS version 9.4 (SAS Institute, Cary, USA).

## Results

### Basic characteristics of the study sample

The present study included 122,502 individuals with IDA and 122,502 individuals without IDA. The basic characteristics of study patients are displayed in Table 1. Mean age was 54.2 [standard deviation (SD) 20.3] years, and 74.1% were women. Patients visited their GPs in average 8.0 times per year during the follow-up. Due to matched pairs design, no significant differences were observable between both cohorts in terms of age, sex, visit frequency, and comorbidities (Table 1).

**Table 1:** Baseline characteristics of the study sample (after propensity score matching)

Variable	Proportion among individuals with iron deficiency anemia (%) N = 122,502	Proportion among individuals without iron deficiency anemia (%) N = 122,502	p value
Age (mean, SD)	54.2 (20.3)	54.2 (20.3)	0.975
Age ≤ 50	47.9	47.3	1.000
Age 51–60	12.3	12.9	
Age 61–70	11.8	11.9	
Age 71–80	15.7	15.6	
Age > 80	12.4	12.3	
Women	74.1	74.1	1.000
Men	25.9	25.9	
Number of physician visits per year during the follow-up (mean, SD)	8.0 (4.0)	8.0 (4.0)	1.000
Diagnoses documented within 12 months prior to or at index date			
Gastrointestinal ulcers	3.8	3.8	1.000
Gastritis	27.9	27.9	1.000
Diabetes	22.1	22.1	1.000
Obesity	13.1	13.1	1.000

Proportions of patients in % given, unless otherwise indicated

*SD* standard deviation

### Association of IDA with subsequent CRC

**Fig. 2:**
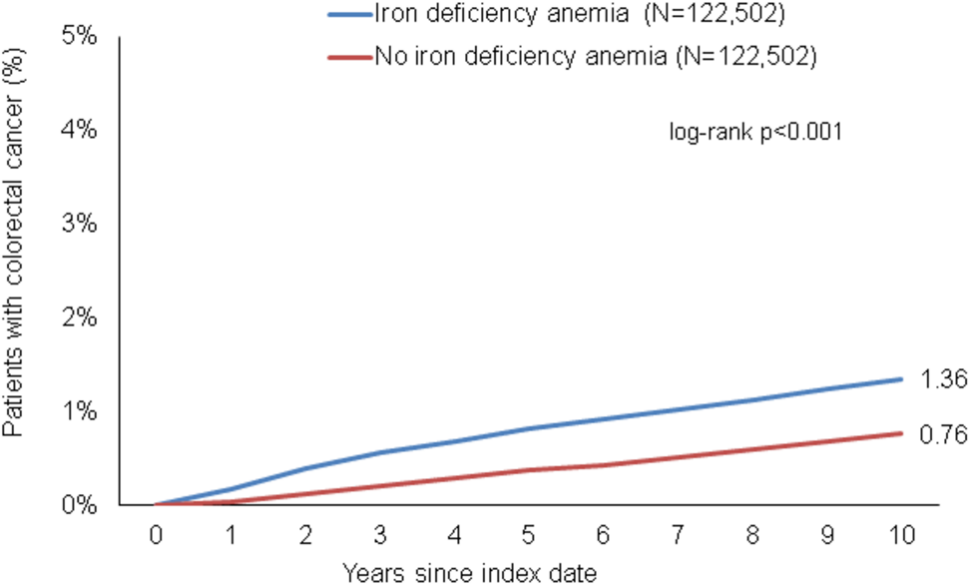
Cumulative incidence of colorectal cancer in individuals with and without iron deficiency anemia

After up to 10 years of follow-up, 1.4% of IDA patients versus 0.8% of matched non-IDA cohort (p < 0.001) were diagnosed with CRC (Fig. 2). In the regression analysis, there was a strong significant association between IDA and subsequent CRC (HR 2.05; 95% CI 1.83–2.30) (Table 2). This association was at strongest in the age group > 80 (HR 3.07; 95% CI 2.39–3.95), and only slightly stronger in men (HR 2.17; 95% CI 1.82–2.59) than in women (HR 2.01; 95% CI 1.73–2.33) (Table 2).

**Table 2:** Association between iron deficiency anemia and subsequent colorectal and stomach cancer in patients followed in general practices in Germany (univariable Cox regression models)

Subcohorts	Incidence (cases per 1000 patients years) in individuals with iron deficiency anemia (%)	Incidence (cases per 1000 patients years) in individuals without iron deficiency anemia (%)	HR (95% CI)	P value
Colorectal cancer				
Total	1.5	0.7	2.05 (1.83–2.30)	< 0.001
Age ≤ 50	0.3	0.2	1.69 (1.20–2.39)	0.003
Age 51–60	1.1	0.7	1.48 (1.07–2.06)	0.018
Age 61–70	2.4	1.1	2.12 (1.63–2.74)	< 0.001
Age 71–80	3.8	1.9	2.00 (1.65–2.41)	< 0.001
Age > 80	5.4	1.7	3.07 (2.39–3.95)	< 0.001
Women	1.2	0.6	2.01 (1.73–2.33)	< 0.001
Men	2.7	1.2	2.17 (1.82–2.59)	< 0.001
Stomach cancer				
Total	0.3	0.2	1.41 (1.13–1.75)	0.002
Age ≤ 50	0.1	0.1	1.16 (0.65–2.08)	0.621
Age 51–60	0.3	0.3	0.98 (0.57–1.70)	0.939
Age 61–70	0.6	0.4	1.42 (0.88–2.28)	0.152
Age 71–80	0.7	0.5	1.34 (0.91–1.97)	0.139
Age > 80	1.1	0.4	2.73 (1.60–4.67)	< 0.001
Women	0.2	0.2	1.08 (0.80–1.46)	0.603
Men	0.8	0.4	1.90 (1.38–2.61)	< 0.001

### Association of IDA with subsequent stomach cancer

**Fig. 3:**
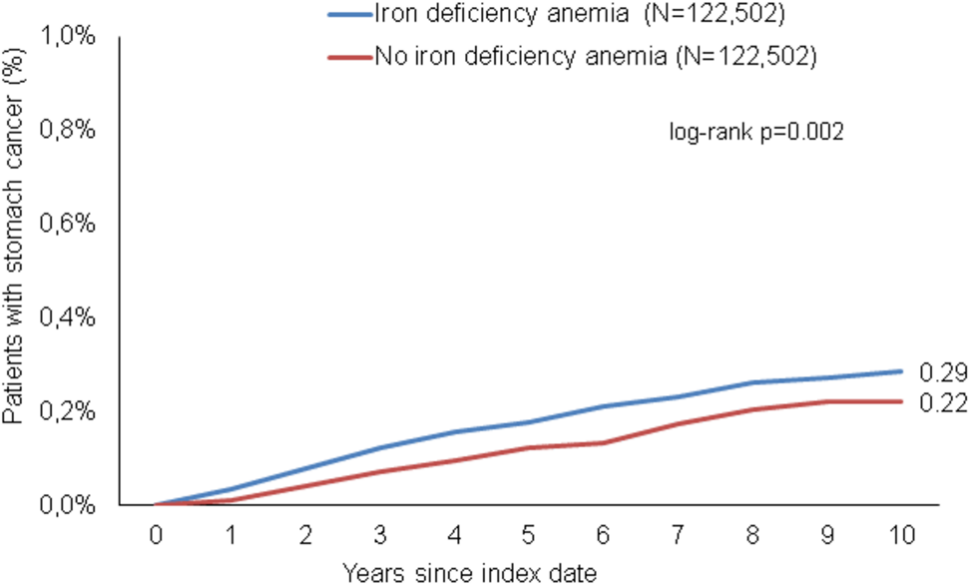
Cumulative incidence of stomach cancer in individuals with and without iron deficiency anemia

After up to 10 years of follow-up, 0.3% of IDA patients versus 0.2% of matched non-IDA cohort (p = 0.002) were diagnosed with stomach cancer (Fig. 3). In the regression analysis, IDA was significantly associated with a subsequent stomach cancer in the total cohorts (HR 1.41; 95% CI 1.13–1.75) (Table 2). However, this association was only strong and significant in the age group > 80 (HR 2.73; 95% CI 1.60–4.67) and men (HR 1.90; 95% CI 1.38–2.61) (Table 2).

## Discussion

This retrospective, population-based study examined the association between IDA and subsequent diagnosis of stomach cancer and CRC in a large German cohort using the IQVIA DA database. Adult patients diagnosed with IDA were compared to a propensity-matched control group of patients without IDA. The results showed that after up to 10 years of follow-up, a total of 1.4% of IDA patients developed CRC compared to 0.8% in the control group. Regression analysis confirmed a strong significant association between IDA and subsequent CRC. This association was strongest in the > 80 age group. Stomach cancer had a 10-year cumulative incidence of 0.3% in IDA patients compared with 0.2% in the non-IDA cohort. However, in the regression analysis, IDA was strongly and significantly associated with subsequent stomach cancer only in the > 80 age group and in men.

Consistent with our findings, a study examining data from the first National Health and Nutrition Examination Survey and Epidemiologic Follow-up Study, including a total of 9024 participants aged 25–74 years, indicated that men and postmenopausal women with ID without anemia had a fivefold and those with IDA a 31-fold increased risk of GI cancer compared with individuals with normal Hb and TSAT levels (Kato et al. 1999). Similarly, Cross et al. demonstrated an inverse association between serum ferritin and TSAT and CRC risk in a Finnish nested case–control study involving a total of 130 CRC patients and 260 controls (Cross et al. 2006). In addition, a retrospective study from California of more than 38,000 male health plan members found a significant association between decreased TSAT and CRC risk (Herrinton et al. 1995). Another case–control study evaluating iron status and CRC in a cohort embedded in the New York University Women's Health Study reported a significant inverse association between serum ferritin and CRC in women in 105 CRC cases and 523 individually matched controls (Kato et al. 1999).

In a retrospective study of data from a single hospital in South Australia examining factors that may increase the risk of neoplasia following a positive fecal immunochemical test (FIT), a subanalysis found that CRC was significantly more common in persons aged ≥ 75 years than in those aged 50–74 years (7.1% vs. 2.1%) and was associated with symptoms (15.8% vs. 1.7%) and IDA (14.6% vs. 2.1%). Interestingly, multivariate analysis showed that IDA and symptoms, but not age, were independent risk factors for CRC following a positive FIT (Hamarneh et al. 2020).

Two cohort studies of Japanese and Japanese Hawaiians have also linked low body iron stores to an increased risk of stomach cancer (Akiba et al. 1991; Nomura et al. 1992). Similarly, Broitman et al. showed an extremely high incidence of more than 90% of preneoplastic gastric lesions without hemorrhage in IDA patients after histologic analysis of 104 patients (Broitman et al. 1981).

In a prospective multicenter study from the United Kingdom, James et al. reported the results of 695 patients hospitalized with suspected IDA. Of note, 11.2% of patients were diagnosed with GI cancer by endoscopy. This incidence was significantly higher than in our study, which may be due to the fact that preselection was performed and only patients who underwent endoscopy were included. In contrast, our data should provide incidences more representative of the general population because the data were collected without preselection in the primary care setting. However, consistent with our observations, James et al. also found that male sex and increasing age, identified in their study as beginning at age 50 years, were significant risk factors for GI cancer in the presence of IDA (James et al. 2005).

The reason for the sex-specific observation and the lack of association between IDA and cancer in premenopausal women may be that both menorrhagia and pregnancy are important causes of IDA in women (James et al. 2005). However, this is unlikely to have a major impact on our results because most of the women in our study were postmenopausal. It is interesting to speculate why these women developed IDA, but dietary ID or postmenopausal gynecologic bleeding should be considered. In fact, menstrual blood loss is the most common cause of IDA in premenopausal women (Annibale et al. 2003) and the prevalence of GI cancer in this group is low (Bini et al. 1998; Ioannou et al. 2002; Annibale et al. 2003; Luman et al. Luman and Ng 2003; Green and Rockey 2004, James et al. 2005).

In addition to our study, a retrospective cohort study from California examined the association between IDA and GI cancer in women aged 20–49 years, including symptoms such as dysphagia, rectal bleeding, and weight loss. The authors noted that upper GI cancer and CRC overall are rare in women of childbearing age and are not affected by the presence or absence of IDA. However, when symptoms rather than IDA were used as an indication for endoscopy, they found the same number of cancers with fewer procedures (Szpakowski and Tucker 2022).

Bidirectional endoscopy appears reasonable for IDA in men and the elderly in the context of cancer diagnosis, but not for premenopausal women with IDA (James et al. 2005). According to the British Society of Gastroenterology (BSG) 2021 recommendation, IDA in young women is not an indication for endoscopy per se, but it is recommended that endoscopy should be performed, particularly depending on the presence of “red flag” symptoms (Snook et al. 2021). Similarly, the American Gastroenterological Association (AGA) Technical Review recommends that patients with GI symptoms should undergo exploratory procedures rather than routine bi-directional endoscopy (Rockey et al. 2020). In contrast, there is high quality indirect evidence from screening studies with randomized controlled trials (RCTs) and non-randomized trials of bidirectional endoscopy in men and postmenopausal women with IDA, showing a significant reduction in mortality and providing confidence in the evidence for the benefit of endoscopic examination in IDA (Brenner et al. 2014; Fitzpatrick-Lewis et al. 2016).

Nevertheless, the age-specific association between IDA and stomach cancer and CRC also observed in our study may reflect the general increase in cancer risk with increasing age. For instance, stomach cancer is known to occur on average at age 71 in men and 76 in women, and CRC at age 72 in men and 75 in women (Barnes et al. 2016). Finally, a possible explanation for the stronger association between IDA and GI cancer found in our study may be the overdiagnosis of IDA in older patients. When these patients are referred for endoscopy to identify possible causes of IDA, they are more likely to be diagnosed with asymptomatic preclinical cancers.

Moreover, iron imbalances appear to have an impact on the oncological course of patients with GI cancer. For example, Zhen et al. investigated the long-term effect of ID on outcome in 644 patients aged 19–83 years with TNM stage II CRC and found that IDA was an independent predictor of long-term outcome in patients with stage T3N0M0 CRC (Zhen et al. 2012). In addition, Lorenzi et al., who examined the survival of 97 patients with CRC in a 5-year follow-up study, found that patients with low or high serum ferritin had shorter survival than patients with normal iron levels (Lorenzi et al. 2006).

Although IDA has been associated with the development of GI tumors in our study and others, the underlying mechanisms are not well understood. Specifically, evidence from preclinical studies suggests that ID attenuates the immune response, allows tumor cells to invade with reduced immune surveillance, and alters the function of immune cells in the tumor microenvironment in favor of the tumor (Muckenthaler et al. 2017; Pfeifhofer-Obermair et al. 2018; Aksan et al. 2021; Phipps et al. 2021). ID also appears to inhibit the expression of several cytokines in cells throughout the immune system (Kuvibidila et al. 2003, 2012; Kuvibidila and Warrier 2004). It is thought that ID causes an imbalance between pro- and antioxidant systems, leading to a decrease in enzymatic and non-enzymatic antioxidant systems and an increase in markers of oxidative stress (Zohora et al. 2018). This in turn leads to increased generation of reactive oxygen species (ROS), which have been linked to tumor cell growth, metastasis, tumor aggressiveness, and resistance to therapy because of their potential to cause cellular damage and DNA mutations (Kobayashi et al. 1988; Koehl et al. 1989; Reuter et al. 2010; Akça et al. 2013; Koskenkorva-Frank et al. 2013; Sies 2015; Gill et al. 2016; Klaunig 2018; Zohora et al. 2018).

However, when interpreting our results, it is important to point out some potential limitations of this study, which are mainly due to the study design and therefore unavoidable. First, the data used in the DA database (IQVIA) is based on the ICD-10 coding system. Therefore, the possibility of miscoding or undercoding of certain diagnoses cannot be excluded. The selection of our sample may also have introduced some bias, as we only used data from GPs in the database and did not include specialties, such as gastroenterology, which are thought to have a higher detection rate for GI cancer. In addition, acute or severe cancers are likely to be treated in hospitals not included in the database. On the other hand, our study avoids referral bias and probably reflects the results of screening the general population for IDA and GI cancer by including all patients diagnosed with IDA and not only those referred for endoscopic examination. In contrast, previous studies investigating the significance of IDA as a marker for occult GI cancer may have overestimated the prevalence of these malignancies in individuals with IDA (Farrell and LaMont 1998), as almost all studies recruited patients referred to gastroenterology for endoscopic examination. Another limitation of the study is that the DA database (IQVIA) does not include information on possible mediating effects, such as lifestyle factors such as smoking, alcohol, diet/nutrition (e.g., vegan or vegetarian lifestyle), and physical activity, or information on socioeconomic status (e.g., education and income), which could confound the association between IDA and cancer risk. It is also important to consider that inadequate iron intake is likely to be associated with deficiencies in several other nutrients or with overall malnutrition, which may have synergistic effects on cancer development. The DA database also does not contain larger panels of laboratory values, including Hb levels and iron parameters, fecal Hb concentrations, or histologic features that would have allowed us to perform further analysis and stratification. There is also no data available on colonoscopy and polypectomy. It should also be noted that only IDA was included in the analysis, not ID without anemia, although ID as such could already have an impact on carcinogenesis. There is also no information on whether and how IDA was treated. In this context, it cannot be determined to what extent iron supplementation (intravenous or oral) may have influenced the present results. It also cannot be excluded that in some cases more iron than necessary was replaced, leading to iron overload, which has already been shown to be associated with an increased risk of cancer (Fonseca-Nunes et al. 2014).

Furthermore, our study is limited by a lack of information on GI symptoms such as dysphagia, abdominal pain, changes in bowel habits, or rectal bleeding. In fact, GI bleeding as a result of GI cancer is a potential cause of IDA. To avoid including patients with IDA due to bleeding as a symptom of GI cancer, cancers diagnosed within 6 months of the index date were excluded from the study. However, it must be acknowledged that a bleeding precancerous lesion may have been present for more than 6 months, leading to chronic blood loss and eventually to IDA. Nevertheless, it should be noted that precancerous lesions are much less likely to bleed than cancers.

Overall, our study cannot make prognostic statements about progression, recurrence, metastasis, or survival and cannot demonstrate causal relationships, only associations.

Despite these limitations, the large sample size and the comparatively long observation period of 10 years should be emphasized as strengths of the study. Furthermore, to our knowledge, our study is the first population-based longitudinal study to investigate the association between IDA and the incidence of stomach and CRC in a German cohort. Finally, cohort matching allowed us to reduce potential confounders and to better estimate the treatment effect of interest.

## Conclusion

Overall, the results of the present study support the findings of other studies indicating that GI cancers are associated with IDA. This association was observed particularly for CRC, in men, and in people over the age of 80. However, the extent to which this association is due to GI bleeding or other pathophysiologic processes that may be caused by IDA cannot be answered by this study. Further investigation of this relationship, particularly to clarify a possible underlying immunological cause of this relationship, in RCTs, especially at the experimental level, should be considered.

## Data Availability

The data that support the findings of this study are available on request from the corresponding author on reasonable request.
